# Accuracy of Artificial Intelligence vs Professionally Translated Discharge Instructions

**DOI:** 10.1001/jamanetworkopen.2025.32312

**Published:** 2025-09-17

**Authors:** Melissa Martos, Blanca Fields, Samuel G. Finlayson, Nigel Hartell, Theresa Kim, Emily Larimer, Jason J. Lau, Yu-Hsiang Lin, Taylor Salaguinto, Nguyen Tran, K. Casey Lion

**Affiliations:** 1 University of Washington, Seattle; 2 Seattle Children’s Hospital, Seattle, Washington; 3 Center for Child Health, Behavior, and Development, Seattle Children's Research Institute, Seattle, Washington

## Abstract

**Importance:**

Patients using languages other than English are a group at risk of poor health outcomes and encounter barriers to access of translated written materials. Although artificial intelligence (AI) may offer an opportunity to improve access, few studies have evaluated the accuracy and safety of AI translation for clinical care under routine practice conditions.

**Objective:**

To investigate the accuracy of AI translation compared with professional human translation of patient-specific issued pediatric inpatient discharge instructions.

**Design, Setting, and Participants:**

This comparative effectiveness analysis compared translations by a neural machine translation model vs professional translators using patient-specific pediatric inpatient discharge instructions received by families between May 18, 2023, and May 18, 2024, at a single center academic pediatric hospital. Instructions were translated to Simplified Chinese, Somali, Spanish, and Vietnamese by professional translators and the Azure AI system and then broken into scoring sections. Two professional translators per language evaluated translations (blinded to source) on an established 5-point scale for fluency, adequacy, meaning, and error severity, with 1 indicating worst performance and 5 indicating best performance.

**Exposure:**

AI vs professional translation.

**Main Outcome and Measure:**

Quality of discharge instruction translation, including fluency, adequacy, meaning, and severity of errors.

**Results:**

A total of 148 sections from 34 discharge instructions were analyzed. When considering all 4 languages together, average fluency, adequacy, and meaning were lower among AI compared with professional human translations. Among all tested languages, mean (SD) fluency for AI translations was 2.98 (1.12) compared with 3.90 (0.96) for professional translations (difference, 0.92; 95% CI, 0.83-1.01; *P* < .001), adequacy was 3.81 (1.14) compared with 4.56 (0.70) (difference, 0.74; 95% CI, 0.65-0.83; *P* < .001), meaning was 3.38 (1.15) compared with 4.28 (0.84) (difference, 0.90; 95% CI, 0.80-0.99; *P* < .001), and error severity was 3.53 (1.28) compared with 4.48 (0.88) (difference, 0.95; 95% CI, 0.85-1.06; *P* < .001). Compared with professional translations, the Spanish AI translations were noninferior in adequacy (difference, 0.08; 95% CI, −0.02 to 0.19) and error severity (difference, 0.03; 95% CI, −0.09 to 0.14) but inferior in fluency (difference, 0.38; 95% CI, 0.23-0.53) and just crossed the inferiority threshold in meaning (difference, 0.08; 95% CI, −0.04 to 0.20). The Chinese, Vietnamese, and Somali AI translations were inferior to the professional translations across all metrics, with the greatest differences for Somali.

**Conclusions and Relevance:**

In this comparative effectiveness analysis of AI- vs professionally translated issued discharge instructions, AI-translated instructions performed similarly for Spanish but worse for other languages tested. Validation and clinical implementation of AI-based translation will require special attention to languages of lesser diffusion to prevent creating new inequities.

## Key Points

**Question:**

How does the accuracy of artificial intelligence (AI)–based translation compare with professional translation of discharge instructions under routine practice conditions?

**Findings:**

In this comparative effectiveness analysis of AI vs professional translation of 148 sections of 34 issued discharge instructions, AI translations were noninferior in some domains for Spanish instructions but consistently inferior in Chinese, Vietnamese, and Somali.

**Meaning:**

These findings suggest that AI translation may have similar performance to professional translations for Spanish discharge instructions but requires further development and validation prior to implementation in other languages.

## Teaser

This comparative effectiveness analysis describes the process of implementation for artificial intelligence–based translation and investigates its accuracy for translating discharge instructions into Simplified Chinese, Somali, Spanish, and Vietnamese.

## Introduction

Approximately 25 million individuals are estimated by the US census to speak English less than “very well,” which is associated with numerous adverse outcomes, including worse satisfaction, comprehension, treatment adherence, and clinical outcomes, higher costs, and more frequent serious safety events.^1,2,3,4,5,6,7,8,9,10,11^ Language barriers affect how care is delivered through both spoken and written communication. Written communication is essential because comprehension of medical information is notoriously low,^12,13,14,15,16,17,18,19,20,21,22,23^ particularly for those who use a language other than English for care and may receive suboptimal verbal communication.^24,25,26,27^ Unfortunately, timely access to professional translation of written materials is lacking due to logistical challenges, cost, and turnaround time. Current systems might deliver translations within 90 to 120 minutes in exceptional circumstances but more commonly require multiple days, limiting access to time-sensitive information.^28^

Modern artificial intelligence (AI)–based models may provide a means of fast, safe, and understandable translation of written materials. However, errors in care instructions can have serious (and potentially dangerous) consequences for patients. Few studies have evaluated the accuracy or safety of AI-based translation of actual issued clinical discharge instructions. Standardized instructions that meet accepted standards for readability, understandability, and completeness differ greatly from actual issued instructions, which frequently fall short.^29,30,31^ Actual clinical discharge instructions are thus likely less accessible to patients of varying health literacy and more difficult to translate.^32,33^ Given that the performance of a translation tool will vary based on the characteristics of the source text, it is critical to test these tools under the conditions in which they would be used. This study described the process of implementation for AI-based translation and investigated its accuracy in translating clinically issued discharge instructions into our top 4 institutional languages.

## Methods

### AI Product Selection and Enrichment

To select a suitable model, we compared 2 forms of AI technologies, a neural machine translation model (Azure AI Translator, Microsoft Corp) and a large language model (Azure Open AI, Microsoft Corp). Neural machine translation models are designed specifically for translating between languages, whereas large language models are designed to create and interpret language more broadly. Our language services team favored the performance of the neural machine translation model during preliminary testing of a set of 2 discharge instructions per language in our top 4 institutional languages other than English (Simplified Chinese, Somali, Spanish, and Vietnamese). Our informatics leaders (who would be involved in future AI translation implementation) also strongly preferred a neural machine translation model due to concern for potential hallucinations with large language models, preferring to err on the side of caution where patient-facing materials were concerned.^34,35,36,37^ The AI interface was built within the Seattle Children’s Hospital tenant. It subsequently underwent enrichment, a standard practice for AI implementation.^38^ Enrichment is critical for ensuring contextual adequacy by providing exposure to specialty-specific terms and languages of lesser diffusion, which may be underrepresented in typical model training datasets.^39^

To enrich the model, professionally translated educational documents, previously created and approved by the Department of Patient Education and Department of Interpreter and Translation Services at the Seattle Children’s Hospital, were provided to the model based on the number available per language (1917 Spanish, 433 Vietnamese, and 216 Simplified Chinese) to enrich the model. We were unable to provide additional training to the Somali model because the vendor had not enabled that option. An initial set of 2 discharge instructions per language were translated by both enriched and unenriched models for Simplified Chinese, Spanish, and Vietnamese. Certified bilingual staff compared the blinded output in detailed written evaluations, determining that only the Vietnamese model performed better after enrichment among the enriched languages. Primary differences related to the handling of formatting and the register of language used, with higher complexity language noted in the enriched model. Thus, the enriched model was used for Vietnamese, whereas base models were used for Simplified Chinese, Somali, and Spanish.

### Discharge Instruction Selection

Patient instructions with a minimum of 75 words issued at Seattle Children’s Hospital between May 18, 2023, and May 18, 2024, were selected. We included only narrative portions of instructions written as free text and/or using 1 or more templated phrases. We did not include prepopulated sections, such as follow-up visit timing or hospital contact information. We used purposive sampling to select a range of diagnoses and specialties and avoid duplicative sampling of the same templated instruction. To achieve this, we began with a consecutive sample, skipping duplicate diagnoses, until we reached our target goal. This study was approved by the Seattle Children’s institutional review board. Informed consent was not required by the institutional review board due to the anonymous nature of the data included. This study follows the International Society for Pharmacoeconomics and Outcomes Research (ISPOR) reporting guideline for comparative effectiveness research, including a priori study design, an explanation of misclassification mitigation, and discussion of internal and external validity of findings.^40^

### Discharge Instruction Translation and Grading

Discharge instructions were classified by medical specialty and degree of personalization, including entirely personalized, entirely templated, or a combination. Each discharge instruction was deidentified and translated into Simplified Chinese, Somali, Spanish, and Vietnamese by professional translators and the AI tool. For scoring, each instruction was broken into sections of approximately 30 to 80 words at contextually logical break points. We sampled discharge instructions until reaching our goal of 148 sections per power calculations, resulting in 34 total instructions. A survey for scoring was built using REDCap (Research Electronic Data Capture),^41,42^ which displayed complete English instructions for context, followed by each scoring section displaying both the English text and its translation, blinded to translation method.

Two translators per language (distinct from those who translated the documents) completed a training block of 6 instruction sets and compared their results, with discussion and resolution of differences to ensure consistency. Afterward, each translator independently scored approximately half of the translated sections. To control for individual variation in grading and minimize the potential impact of misclassification, the AI and professional versions of the same instruction were always scored by the same translator (not in immediate succession).

Scoring was performed for the following 4 domains, which are established metrics for evaluating translation accuracy: fluency, adequacy, meaning, and severity of errors.^43,44^ Fluency referred to clarity and flow of writing, including appropriate vocabulary and grammar. Adequacy referred to accuracy, completeness, and appropriateness for the context. Meaning referred to maintenance of the substance of the original text, including connotation and semantics. Severity of errors referred to the expected impact errors may have on clinical care, with 1 indicating worst performance and 5 indicating best performance. Error severity was classified as clinically impactful if ranked 1 to 3 (see Table 1 for examples). Errors marked by translators as “unsure of clinical impact” were rescored by 2 pediatricians (M.M., E.L., J.J.L., and/or K.C.L.), with disagreements resolved by discussion to reach consensus. For a detailed scoring guide, see the eMethods in Supplement 1.

**Table 1.** Example Scoring for Severity of Errors^a^

Score	Source text	Error
Clinically impactful		
1 (Harms patient)	“Yellow Zone: I do not feel good. I need to take rescue medications and my controller medicines listed below to keep from getting worse.”	“Yellow Zone: I do not feel good. [omitted].”
	“Always check to make sure the [nasogastric] tube is in the right place before each feeding.”	“Always check to make sure the test tube is in the right place before each feeding.”
2 (Impairs care)	“What can I expect after surgery? Changes in sensation.”	“What can I expect after surgery? Changes in poetry.”
3 (Delays care)	“Call the cardiologist on-call for any of the following: limping on the leg(s) of catheter site(s)”	“Call the cardiologist on-call for any of the following: [unintelligible] on the leg(s) of catheter site(s)”
Not clinically impactful		
4 (Unclear effect on patient care)	“Call your Primary Healthcare Provider for any of the following: lethargy”	“Call your Primary Healthcare Provider for any of the following: excessive sleeping”
5 (No effect on patient care)	*“*Call the Orthopedic Resident on-call at 206-987-2000 (Evenings/Nights/Weekends)”	*“*Call the Orthopedic Resident [omitted] at 206-987-2000 (Evenings/Nights/Weekends)”

^a^ Discharge instructions were scored by professional translators blinded to source in 4 languages. Scores were assigned on a scale of 1 to 5, with 1 indicating most harmful (harms patient) and 4 to 5 indicating not clinically impactful.

### Statistical Analysis

We conducted a comparative effectiveness study comparing the mean scores for AI translation with the mean scores for professional translation in each of the 4 domains. Means were compared within each language using paired *t* tests. Our noninferiority limit was set to a difference of less than 0.2 on the 5-point Likert scale (a 4% absolute difference), determined in collaboration with our language services colleagues as a clinically meaningful threshold. The CIs for the difference in means that included this margin of 0.2 or greater were interpreted as evidence of inferiority. Proportions of error severity scores deemed clinically impactful within each language were also compared using the paired Wilcoxon signed-rank test. We also compared the proportion of overall instructions with clinically impactful errors within each language by the McNemar test. *P* < .05 was considered statistically significant. Assuming an SD of 0.7 as suggested by prior work,^45^ α = .05, and a power of 0.80, the study was adequately powered with 148 translated sections to detect a difference of 0.2 or more.

## Results

A total of 34 discharge instructions were identified and broken into 148 scoring sections. Complete discharge instruction length ranged from 76 to 1177 words, with a median of 243 words. Pediatric medical specialties were represented in 22 (65%) and surgical in 12 (35%); among medical specialties, 15 (68%) were from general pediatrics, whereas the remainder were from subspecialty services (Table 2).

**Table 2.** Characteristics of the Issued Discharge Instructions Taken From Seattle Children’s Hospital Between 2023 and 2024

Characteristic	No. (%) of discharge instructions (N = 34)
Field	
Medical	22 (65)
Cardiology	1 (3)
General pediatrics	15 (44)
Hematology-oncology	1 (3)
Neonatal intensive care	2 (6)
Rehabilitation	2 (6)
Rheumatology	1 (3)
Surgical	12 (35)
Cardiac surgery	1 (3)
General surgery	3 (9)
Oral and maxillofacial surgery	2 (6)
Orthopedics	3 (9)
Plastic and craniofacial surgery	1 (3)
Transplant surgery	1 (3)
Urology	1 (3)
Type of instructions	
Free texted	6 (18)
Combination of free texted and templated	1 (3)
Templated	27 (79)

When considering all 4 languages together, mean fluency, adequacy, and meaning were lower among AI compared with professional human translations. Among all tested languages, mean (SD) fluency for AI translations was 2.98 (1.12) compared with 3.90 (0.96) for professional translations (difference, 0.92; 95% CI, 0.83-1.01; *P* < .001), adequacy was 3.81 (1.14) compared with 4.56 (0.70) (difference, 0.74; 95% CI, 0.65-0.83; *P* < .001), meaning was 3.38 (1.15) compared with 4.28 (0.84) (difference, 0.90; 95% CI, 0.80-0.99; *P* < .001), and error severity was 3.53 (1.28) compared with 4.48 (0.88) (difference, 0.95; 95% CI, 0.85-1.06; *P* < .001).

**Figure 1.**
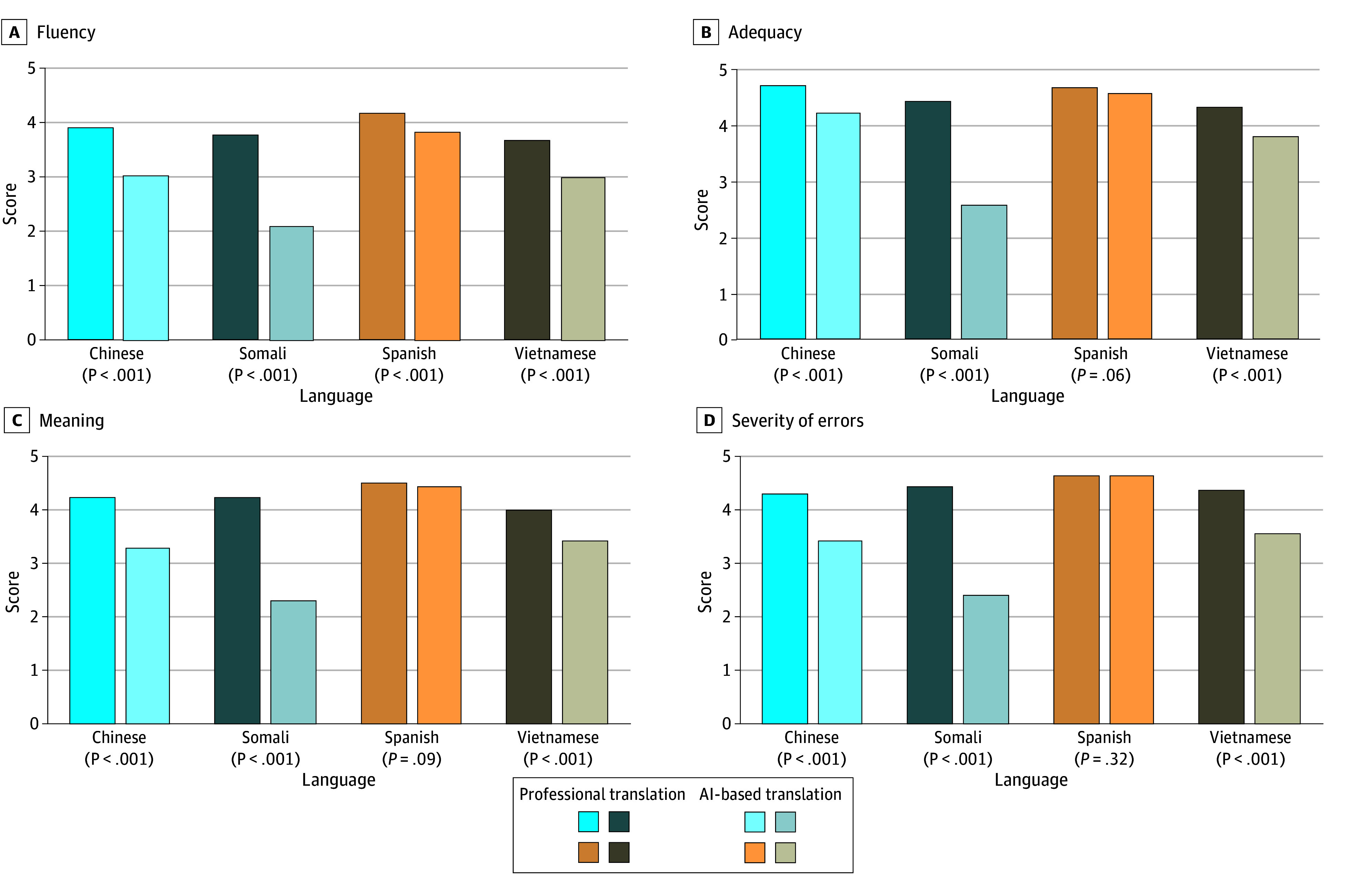
Comparison of Mean Fluency, Adequacy, Meaning, and Error Severity Scores for Professionally Translated vs Artificial Intelligence (AI)–Translated Discharge Instructions Discharge instructions (n = 148 for each category) were scored by professional translators blinded to source in 4 languages. Scores were assigned on a scale of 1 to 5, with 1 indicating worst performance and 5 indicating best performance. *P* values are based on paired *t* tests testing for differences between professional and AI translation of issued discharge instructions (rather than noninferiority). Significant differences were found between professional and AI translations in all categories for all languages other than Spanish, where the only significant difference was found in fluency.

AI performance varied based on language tested (Figure 1 and Table 3). In Spanish, AI translations were noninferior in adequacy and error severity, but fluency (3.82 vs 4.20; difference, 0.38; 95% CI, 0.23-0.53; *P* < .001) was significantly worse for AI translations compared with professional translations. Meaning was not significantly different between Spanish AI and professional translations, but AI translation failed to meet the noninferiority threshold with a CI at the limit of 0.20 (4.46 vs 4.54 for AI vs professional; difference, 0.08; 95% CI, −0.04 to 0.20). Meanwhile, AI translations were inferior in every category for Simplified Chinese, Somali, and Vietnamese (Table 3). In Simplified Chinese, AI translations performed consistently worse than professional translations, including for fluency (difference, 0.89; 95% CI, 0.72-1.06), adequacy (difference, 0.49, 95% CI, 0.34-0.64), meaning (difference, 0.95, 95% CI, 0.77-1.14), and error severity (difference, 0.91, 95% CI, 0.69-1.12), all significant (*P* < .001 for all); similar differences were found between AI and professional translations in Vietnamese (fluency: difference, 0.70; 95% CI, 0.55-0.84; adequacy: difference, 0.54; 95% CI, 0.40-0.68; meaning: difference, 0.59; 95% CI, 0.42-0.76; error severity: difference, 0.83; 95% CI, 0.66-1.00; *P* < .001 for all). AI translations performed worst in Somali, where there were significant and large differences in every category, including fluency (difference, 1.71; 95% CI, 1.53-1.89), adequacy (difference, 1.86; 95% CI, 1.68-2.03), meaning (difference, 1.97; 95% CI, 1.80-2.13), and error severity (difference, 2.05; 95% CI, 1.86-2.23; *P* < .001 for all).

**Table 3.** Mean Fluency, Adequacy, Meaning, and Error Severity Scores for Professionally Translated vs AI-Translated Discharge Instructions

Variable	Mean score (95% CI)			
	Fluency	Adequacy	Meaning	Error severity
Simplified Chinese				
Professional	3.91 (3.76 to 4.06)	4.72 (4.61 to 4.82)	4.26 (4.12 to 4.41)	4.35 (4.19 to 4.51)
AI	3.02 (2.84 to 3.20)	4.23 (4.06 to 4.40)	3.31 (3.13 to 3.49)	3.45 (3.25 to 3.64)
Difference	0.89 (0.72 to 1.06)	0.49 (0.34 to 0.64)	0.95 (0.77 to 1.14)	0.91 (0.69 to 1.12)
* P* value^a^	<.001	<.001	<.001	<.001
Somali				
Professional	3.79 (3.65 to 3.94)	4.45 (4.33 to 4.58)	4.28 (4.14 to 4.41)	4.47 (4.34 to 4.60)
AI	2.08 (1.98 to 2.18)	2.59 (2.45 to 2.74)	2.31 (2.19 to 2.43)	2.43 (2.29 to 2.57)
Difference	1.71 (1.53 to 1.89)	1.86 (1.68 to 2.03)	1.97 (1.80 to 2.13)	2.05 (1.86 to 2.23)
*P* value^a^	<.001	<.001	<.001	<.001
Spanish				
Professional	4.20 (4.05 to 4.35)	4.70 (4.59 to 4.80)	4.54 (4.43 to 4.65)	4.69 (4.57 to 4.81)
AI	3.82 (3.68 to 3.97)	4.61 (4.52 to 4.71)	4.46 (4.35 to 4.57)	4.66 (4.56 to 4.77)
Difference	0.38 (0.23 to 0.53)	0.08 (−0.02 to 0.19)	0.08 (−0.04 to 0.20)	0.03 (−0.09 to 0.14)
* P* value^a^	<.001	.06	.09	.32
Vietnamese				
Professional	3.70 (3.54 to 3.87)	4.36 (4.24 to 4.47)	4.03 (3.90 to 4.17)	4.42 (4.26 to 4.57)
AI	3.01 (2.84 to 3.18)	3.82 (3.68 to 3.95)	3.45 (3.31 to 3.59)	3.59 (3.39 to 3.78)
Difference	0.70 (0.55 to 0.84)	0.54 (0.40 to 0.68)	0.59 (0.42 to 0.76)	0.83 (0.66 to 1.00)
* P* value^a^	<.001	<.001	<.001	<.001

Abbreviation: AI, artificial intelligence.

^a^ *P* values are based on paired *t* tests comparing professional and AI translation of issued discharge instructions.

**Figure 2.**
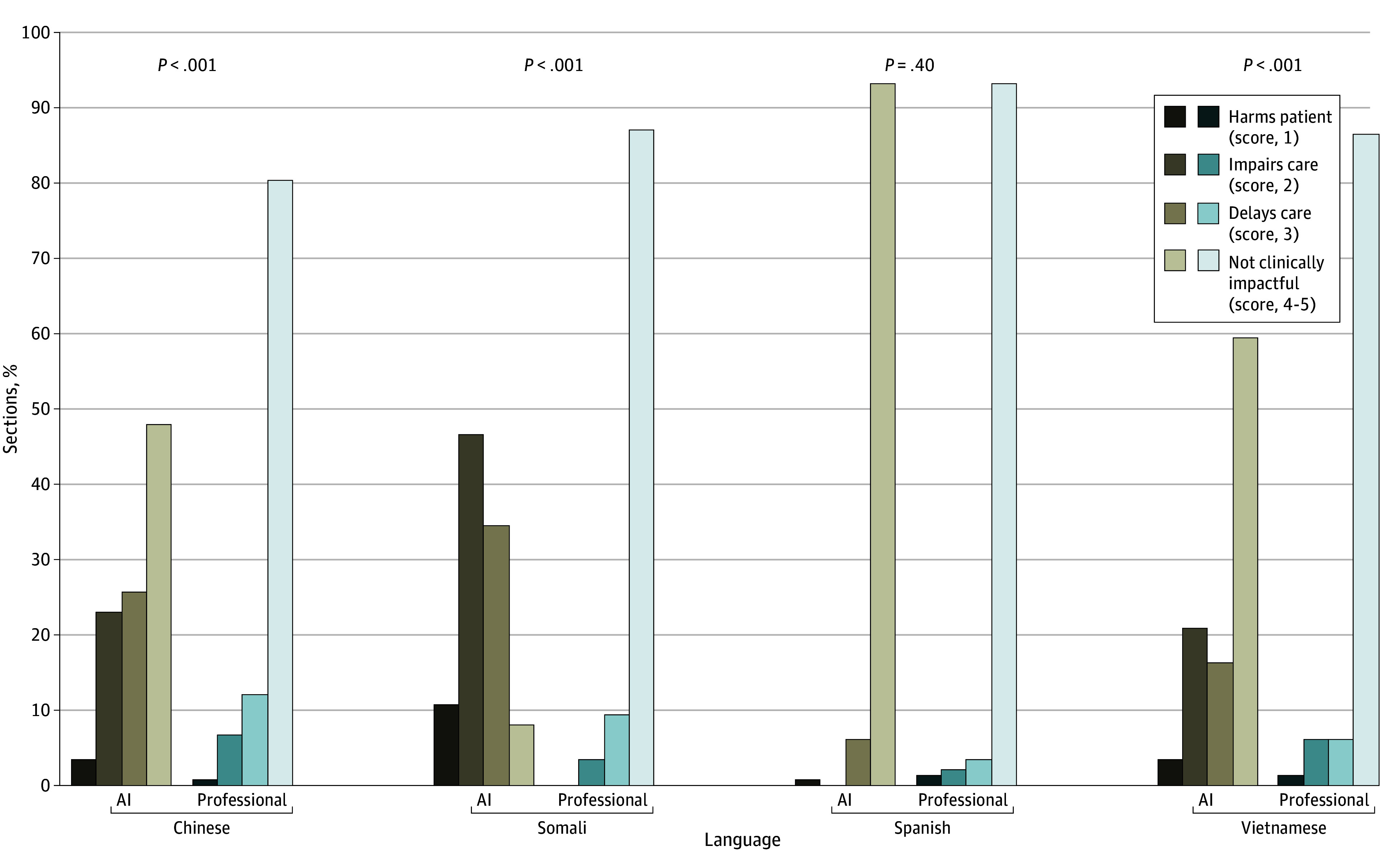
Frequency of Clinically Impactful Errors by Language and Translation Type Discharge instructions were scored by professional translators blinded to source in 4 languages (n = 148 per category). Scores were assigned on a scale of 1 to 5, with 1 indicating most harmful (harms patient) and 4 to 5 indicating not clinically impactful. *P* values are based on paired Wilcoxon signed-rank testing for significant differences between professional and artificial intelligence (AI) translation of issued discharge instructions (rather than noninferiority). No significant differences were found in proportions of errors in Spanish, but all other groups showed significantly worse performance of the AI.

The numbers of clinically impactful errors (Table 1) were also significantly greater in AI than in professional translations in every language other than Spanish (Figure 2). In Spanish, only 10 sections (7%) in both AI and professional translations (*z* = 0.84, *P* = .40) had errors that were clinically impactful, whereas in Somali, 136 (92%) (AI) vs 19 (13%) (professional) (z = 10.43, *P* < .001) were clinically impactful. Differences were also significant in Simplified Chinese (77 [52%] vs 29 [20%], *z* = 7.17, *P* < .001) and Vietnamese (60 [41%] vs 20 [14%], z = 7.96, *P* < .001). When comparing errors at the level of the overall instruction set, AI-translated instructions were significantly more likely to have clinically impactful errors compared with human translations in Chinese (30 [88.2%] vs 20 [58.8%], *P* = .004), Somali (33 [97.1%] vs 11 [32.4%], *P* < .001), and Vietnamese (28 [82.4%] vs 13 [38.2%], *P* < .001); there was no difference for Spanish (8 [23.5%] vs 4 [11.8%], *P* = .22) (eTable in Supplement 1). Notably, this analysis does not account for differences in length of each instruction set.

## Discussion

In this study comparing AI-based vs professional human translations in 4 languages, our findings reveal that although the AI model produced translated discharge instructions that were largely comparable to professionally translated ones in Spanish, the AI translations were inferior across the board in Simplified Chinese, Somali, and Vietnamese. In Spanish, AI translations were noninferior in adequacy and error severity but inferior in meaning; AI translations were inferior in fluency for all languages tested. Our findings also revealed a high proportion of clinically impactful errors for translations in languages other than Spanish, highlighting the need for caution when considering AI implementation in medical settings in its current form. These results align with prior studies,^45,46,47^ which demonstrate fair accuracy for prevalent languages but notable underperformance for those of lesser diffusion.

The body of literature on AI-based translation for medical care is limited but rapidly expanding. Prior studies^43,45^ have highlighted the potential utility of AI models for translating discharge instructions. Other work^48,49^ has investigated models applied to other translation needs, such as medication instructions and educational information. Notably, most studies have evaluated translations of standardized, curated content,^45,50^ which may not fully reflect the complexities of documents in clinical practice. Our study makes an important contribution to the literature by examining AI performance on actual issued clinical discharge instructions, using a robust, fully powered sample to evaluate performance across multiple languages. Furthermore, although other studies^43,45^ have used volunteer native speakers or bilingual physicians for evaluation, we engaged professional translators, whose linguistic expertise may offer particularly discerning assessment. Consistent with previous findings, our study underscores both the promise of these models and the ongoing need for improvement.

Medical AI translation technology is evolving rapidly. Section 1557 of the Patient Protection and Affordable Care Act prohibits automated translation of medical documents without human review “when accuracy is essential,” “when the source documents or materials contain complex, nonliteral or technical language,” or “when the underlying text is critical to the rights, benefits, or meaningful access” of individuals using a language other than English for care.^51,52^ However, as AI models improve, less time and effort may be needed for human review and editing. The need for rapid-turnaround written document translation continues to increase as more platforms, such as patient portals, become central to care delivery and traditional means of communication become less supported. AI translation may offer an opportunity to address these key areas of need, but differences in context, language conventions, and formatting in each scenario require tailored implementation.

Of particular importance is ensuring that the technology evolves to support languages of lesser diffusion and those linguistically different from English. Our model’s performance in Spanish was similar to that of professional translators, but that was not the case for the other languages tested; others have found similar patterns.^45,46,47^ Worse performance of machine translation is a known phenomenon when working between languages that are syntactically different, such as English and Chinese.^53,54^ Furthermore, less availability of quality translated training documents available for languages of lesser diffusion presents a challenge for quality model output in these languages.^39,55^ Prevalent languages in the US, such as Spanish, have more infrastructure to support them, including the widespread availability of interpreters and translated education materials, websites, and prescriptions.^56,57,58,59^ The addition of AI supporting language access in Spanish is important, but gaps in access are most profound among languages of lesser diffusion, which may present the greatest potential for important breakthroughs in health care equity. There is great promise for technology to improve the care for patients using a language other than English, but as we implement new technology we must make concerted efforts to include patients from languages less prevalent locally. Rather than settling for lower-quality translations in these languages or excluding them altogether, we must prioritize enhancing AI performance in these other languages to ensure equitable language access.

How can we improve AI performance to serve patients and families using all languages? First, we can better train AI models to navigate specific uses in diverse languages on both the development and user (eg, hospital system) sides. Further development of translated written materials and methods to overcome inadequate training materials for languages of lesser diffusion is essential.^55,60,61,62^ Our efforts to enrich the translation models using local materials provided useful insights, most notably that enrichment did not always improve model performance, highlighting the need for evaluation prior to implementation. Nonetheless, it is essential that vendors offer the ability to locally enrich models in every language for equitable access to AI resources. Limited professionally translated local enrichment materials also form a barrier for less prevalent languages.^63^ Enrichment materials should be thoughtfully selected to ensure that they are clearly written for patients and families so that output translations will be understandable. Next, once an AI model is implemented, rigorous monitoring by an overseeing body must ensure that output quality does not decrease with time, a phenomenon known as dataset shift that occurs due to differences between actual and training data.^64^ Lastly, we can improve partnerships across institutions and vendors to avoid “reinventing the wheel” at each institution. Because AI models are generally proprietary technology, our ability to collaborate is often limited. We can support improvement across the board by openly sharing professionally translated enrichment documents and training sets. Such sharing will require partnerships among institutions and navigation of content ownership that ensures that translators who have contributed their intellectual property are appropriately compensated for their efforts. Through greater collaboration, we can take advantage of market diversity to improve technology and better serve patients and families using all languages.

As AI translation technology advances, its potential to improve patient care increases; however, careful evaluation is essential to avoid unintended consequences that could deepen inequities. Innovations must be introduced thoughtfully, focusing on understanding the experiences and needs of the families they aim to support. While pursuing progress, we must avoid making assumptions about how families prioritize translation quality versus speed. Achieving equitable and effective outcomes will require patient and family input at every stage of AI implementation.

### Limitations

Our study had limitations. As with any study investigating AI implementation in medicine, the validity of the results applies to the specific AI model used, and models are rapidly changing. Furthermore, enriching an AI model to address a specific context adequately^38^ leads to further differentiation of models, making reproducibility challenging. However, it is reassuring that our results are consistent with others using other models. Although it can be hypothesized that similarly less common languages may perform less well, our results cannot be generalized to languages other than those tested. Further work exploring AI translation accuracy in other contexts and adult settings may also be informative. Despite blinding, translators may have been able to detect when translations were created by the AI due to nuances in language and fluency. Additionally, there may have been differences in scoring between individual translators; however, each translator scored both the AI and professional translations for that language. Thus, although conclusions that can be drawn about differences between languages are limited, differences within languages are meaningful. Additionally, we used a professional vendor for human translations to ensure they were representative of a standard approach and thus do not have access to data comparing time required for professional vs AI translations.

## Conclusions

In this comparative effectiveness analysis of blinded translator review of AI vs professional translation of 148 sections of actual discharge instructions into 4 languages, our AI model produced translations that were largely noninferior to professional translations for Spanish but underperformed in all other languages tested, consistent with prior literature. The results included a high proportion of clinically impactful errors in AI translations. Although current models may perform well for translating discharge instructions for widely spoken languages, considerable work remains to improve performance for languages that are less prevalent in the US. Thoughtful and inclusive implementation of this technology may lead to more equitable care for patients and families.

## Notes

Supplement 1. **eMethods.** Translation Scoring Guide **eTable.** Proportion of Issued Discharge Instructions With Clinical Impactful Errors, Comparing Professional to AI Translations

Supplement 2.
Data Sharing Statement
