# Supplementary material for: Accuracy of Artificial Intelligence vs Professionally Translated Discharge Instructions
Source: JAMA Netw Open. 2025 Sep 17;8(9):e2532312. doi: 10.1001/jamanetworkopen.2025.32312 (PMC12444566; doi:10.1001/jamanetworkopen.2025.32312)
Supplement: Supplement 1. — eMethods. Translation Scoring Guide eTable. Proportion of Issued Discharge Instructions With Clinical Impactful Errors, Comparing Professional to AI Translations [file jamanetwopen-e2532312-s001.pdf]

Supplemental Online Content

Martos M, Fields B, Finlayson SG, et al. Accuracy of artificial intelligence vs professionally translated discharge instructions. *JAMA Netw Open*. 2025;8(9):e2532312. doi:10.1001/jamanetworkopen.2025.32312

eMethods. Translation Scoring Guide

eTable. Proportion of Issued Discharge Instructions With Clinical Impactful Errors, Comparing Professional to AI Translations

This supplemental material has been provided by the authors to give readers additional information about their work.

© 2025 Martos et al. *JAMA Network Open.*

AI-Based Translation- Scoring Guide

Scoring Domains

Fluency

Written smoothly, ensuring that the text is easy to read and understand.

- Clear Expression
- Proper Grammar
- Appropriate Vocabulary
- Logical Flow

1	No fluency; no appreciable grammar, not understandable
2	Marginal fluency; several grammatical errors
3	Good fluency; several grammatical errors, understandable
4	Excellent fluency; few grammatical errors
5	Perfect fluency; like reading a newspaper

Adequacy

Extent to which the writing meets the required standards or expectations for the context (I.e., discharge instructions).

- Relevance
- Clarity
- Accuracy
- Completeness

1	0% of information conveyed from the original
2	25% of information conveyed from the original
3	50% of information conveyed from the original
4	75% of information conveyed from the original
5	100% of information conveyed from the original

Meaning

Significance or interpretation of words, sentences, and texts.

- Connotation.
- Context
- Syntax
- Semantics

1	Totally different meaning from the original
2	Misleading information added/omitted compared to the original
3	Partially the same meaning as the original
4	Almost the same meaning as the original
5	Same meaning as the original

Severity (of errors)

Impact that mistakes have on the clarity, readability, and overall quality of the writing. For this domain, think about how the errors could impact their clinical care.

1	Dangerous to patient
2	Impairs care in some way
3	Delays necessary care
4	Unclear effect on patient care
5	No effect on patient care
6	Unsure. Provide details:

Open Response

Each excerpt will have a text box where details, context, or notes can be added.

© 2025 Martos et al. *JAMA Network Open.*

Example Translation Review

For each excerpt you will be reviewing the English will be to the left, with the non-English translation on the right.
You will be blinded to whether or not the translation was completed by a professional translator or AI.

Spanish

English	Spanish Translation
**Instructions**<br>**Leptomeningeal Glioneuronal Tumor**<br>**Neurosurgery**<br>• For non-urgent questions, call Neurosurgery Clinic at 206-987-xxxx, option 4. Your call will be returned within 1 business day.<br>• For urgent questions and any of the below symptoms, call the Hospital at 206-987-xxxx and ask to page the Neurosurgery Provider on-call.	**Instrucciones**<br>**Tumor glioneuronal leptomeníngeo**<br>**Neurocirugía**<br>• Para preguntas no urgentes, llame a Neurocirugía al 206-987-xxxx, opción 4. Se regresará su llamada dentro de 1 hábil.<br>• Para preguntas urgentes y cualquiera de los siguientes síntomas, llame a la central telefónica del hospital al 206-987-xxxx y pida por el proveedor de Neurocirugía de guardia.

How would you score the translation in the following domains?

Fluency | Adequacy | Meaning | Severity (of errors)

© 2025 Martos et al. *JAMA Network Open.*

Seattle Children's®
HOSPITAL • RESEARCH • FOUNDATION

eTable 1: Proportion of issued discharge instructions with clinically impactful errors, comparing professional to AI translations.

	N (%) Translated Discharge Instruction Sets with a Clinically Impactful Error			
	Professional Translations	AI Translations	*p*-value	χ^2 (df)
**Simplified Chinese**	20 (58.8)	30 (88.2)	**.004**	8.1 (1)
**Somali**	11 (32.4)	33 (97.1)	**<0.001**	20.0 (1)
**Spanish**	4 (11.8)	8 (23.5)	.22	1.5 (1)
**Vietnamese**	13 (38.2)	28 (82.4)	**<0.001**	13.1 (1)

Discharge instructions were scored by professional translators blinded to source in 4 languages. Scores were assigned on a scale from 1 to 5, with 1 being most harmful (harms patient), 1-3 clinically impactful, and 4-5 not clinically impactful. For this table, entire instruction sets (not instruction sections) were compared within each language group. P-values are based on paired McNemar's tests comparing professional and AI translations. Significant p values are bolded.
