# Supplementary material for: Accuracy of Artificial Intelligence vs Professionally Translated Discharge Instructions
Source: JAMA Netw Open. 2025 Sep 17;8(9):e2532312. doi: 10.1001/jamanetworkopen.2025.32312 (PMC12444566; doi:10.1001/jamanetworkopen.2025.32312)
Supplement: Supplement 2. — Data Sharing Statement [file jamanetwopen-e2532312-s002.pdf]

Data Sharing Statement

Martos. Accuracy of Artificial Intelligence vs Professionally Translated Discharge Instructions. *JAMA Netw Open*. Published September 17, 2025. doi:10.1001/jamanetworkopen.2025.32312

Data
Data available: Yes
Data types: Other (please specify)
Additional Information: de-identified dataset and data dictionary
How to access data: Researchers from other institutions will have the option to contact us directly at melissa.martos@seattlechildrens.org for a fully de-identified dataset. These data will be made available only if users agree to a data sharing plan that asserts their commitment to: 1) using data solely for research purposes, 2) accessing the data through a secure server and complying with all HIPPA regulations for appropriately safeguarding data, and 3) destroying the data upon completion of their proposed project.
When available: With publication

Supporting Documents
Document types: None

Additional Information
Who can access the data: These data will be made available only if users agree to a data sharing plan that asserts their commitment to: 1) using data solely for research purposes, 2) accessing the data through a secure server and complying with all HIPPA regulations for appropriately safeguarding data, and 3) destroying the data upon completion of their proposed project.
Types of analyses: These data will be made available for any purpose with approval of a proposal as above.
Mechanisms of data availability: Data will be made available with approval of a proposal.
